# Leucine elicits myotube hypertrophy and enhances maximal contractile force in tissue engineered skeletal muscle in vitro

**DOI:** 10.1002/jcp.25960

**Published:** 2017-05-08

**Authors:** Neil R.W. Martin, Mark C. Turner, Robert Farrington, Darren J. Player, Mark P. Lewis

**Affiliations:** ^1^ School of Sport, Exercise and Health Sciences Loughborough University Loughborough UK; ^2^ National Centre for Sport and Exercise Medicine, School of Sport, Exercise and Health Sciences Loughborough University Loughborough UK; ^3^ Arthritis Research UK Centre for Sport, Exercise and Osteoarthritis, School of Sport, Exercise and Health Sciences Loughborough University Loughborough UK

**Keywords:** amino acids, hypertrophy, mTORC1, skeletal muscle

## Abstract

The amino acid leucine is thought to be important for skeletal muscle growth by virtue of its ability to acutely activate mTORC1 and enhance muscle protein synthesis, yet little data exist regarding its impact on skeletal muscle size and its ability to produce force. We utilized a tissue engineering approach in order to test whether supplementing culture medium with leucine could enhance mTORC1 signaling, myotube growth, and muscle function. Phosphorylation of the mTORC1 target proteins 4EBP‐1 and rpS6 and myotube hypertrophy appeared to occur in a dose dependent manner, with 5 and 20 mM of leucine inducing similar effects, which were greater than those seen with 1 mM. Maximal contractile force was also elevated with leucine supplementation; however, although this did not appear to be enhanced with increasing leucine doses, this effect was completely ablated by co‐incubation with the mTOR inhibitor rapamycin, showing that the augmented force production in the presence of leucine was mTOR sensitive. Finally, by using electrical stimulation to induce chronic (24 hr) contraction of engineered skeletal muscle constructs, we were able to show that the effects of leucine and muscle contraction are additive, since the two stimuli had cumulative effects on maximal contractile force production. These results extend our current knowledge of the efficacy of leucine as an anabolic nutritional aid showing for the first time that leucine supplementation may augment skeletal muscle functional capacity, and furthermore validates the use of engineered skeletal muscle for highly‐controlled investigations into nutritional regulation of muscle physiology.

## 1 INTRODUCTION

Skeletal muscle growth is regulated primarily by the mammalian target of rapamycin complex 1 (mTORC1) signaling pathway, which enhances the capacity for mRNA translation and reduces flux through catabolic pathways such as the autophagy‐lysosome and the ubiquitin proteasome system (Nicklin et al., 2009). mTORC1 signaling has consistently shown to be activated in response to both muscle loading (e.g., resistance exercise), and amino acid consumption/treatment (Marcotte, West, & Baar, 2015), and as such these stimuli represent excellent candidates as therapies for attenuating the muscle wasting associated with a number of disease states and ageing. Indeed, acute human studies have observed activation of mTORC1 and its downstream targets (e.g., p70S6K, rpS6, and 4EBP‐1) following ingestion of mixed amino acids, and this is coupled with an increase in muscle protein synthesis (MPS) in the ensuing 60–120 min (Atherton, Etheridge et al., 2010; Koopman et al., 2006; Paddon‐Jones et al., 2004; Volpi, Kobayashi, Sheffield‐Moore, Mittendorfer, & Wolfe, 2003). Furthermore, when human skeletal muscle undergoes loading prior to amino acid ingestion this effect on mTORC1 signaling and MPS is potentiated (Moore, Atherton, Rennie, Tarnopolsky, & Phillips, 2011; Witard et al., 2014). The necessity for mTOR activation in mediating the MPS response to both amino acids and muscle loading is evidenced by the fact that in rodents, both stimuli fail to augment the synthetic response when in the presence of the mTOR inhibitor rapamycin (Anthony et al., 2000; Kubica, Bolster, Farrell, Kimball, & Jefferson, 2005).

The anabolic properties of amino acid ingestion have been largely attributed to the essential amino acids, and in particular the branched chain amino acid leucine. A number of lines of research support this notion; first, whey protein, which has a high leucine content results in superior MPS rates in humans compared to soy or casein, which have lower leucine contents (Tang, Moore, Kujbida, Tarnopolsky, & Phillips, 2009). Second, ingestion of small quantities of leucine rich essential amino acids activate the downstream mTORC1 target p70S6k and MPS in a comparable manner to 20–25 g of whey protein, and to a greater extent than a bolus of leucine‐deficient essential amino acids (Bukhari et al., 2015; Churchward‐Venne et al., 2012), and removal of leucine from an essential amino acid supplement following muscle loading attenuates mTORC1 signaling (Moberg et al., 2014). Finally, in C2C12 myotubes in vitro, leucine exhibits the most potent stimulation of mTORC1 signaling compared to all other amino acids (Atherton, Smith, Etheridge, Rankin, & Rennie, 2010), and its deprivation impairs protein synthesis and phosphorylation of p70S6k (Talvas, Obled, Fafournoux, & Mordier, 2006).

In vitro cultures of skeletal muscle provide a controlled and isolated environment in which to understand cellular and molecular adaptation, and have improved our understanding of the importance of amino acids, and in particular leucine, for skeletal muscle growth (Areta, Hawley, Ye, Chan, & Coffey, 2014; Atherton, Smith et al., 2010; Talvas et al., 2006). However, a limitation of conventional in vitro methods is the inability of the rigid 2‐dimensional substrate to support muscle contraction, and as such only acute experiments are typically possible. Tissue engineered skeletal muscle however allows for skeletal muscle progenitor cells to be cultured on/inside biologically relevant substrates in 3‐dimensions, and are less stiff, in turn supporting improvements in levels of skeletal muscle maturation (Engler et al., 2004), and generation of contractile force. Indeed, the ability to stimulate and measure contractile force within tissue engineered skeletal muscle is well reported (Cheng, Davis, Madden, Bursac, & Truskey, 2014), and while we and others (Martin et al., 2015; Ostrovidov et al., 2017) have made efforts toward increasing the biological accuracy of such tissues, the removal of interfacing cell types (e.g., motor neurons) allows for muscle specific effects of a given intervention to be explored. As such, engineered skeletal muscle provides an ideal screening platform in which to better understand the impact of leucine supplementation on muscle size and function in an in vitro setting that closely replicates native skeletal muscle architecture and function.

In the present study, we therefore aimed to determine if leucine supplementation would enhance contractile force and myotube size in engineered skeletal muscle, and whether this phenomenon may be dose‐dependent. Furthermore, we sought to determine the role of the mTOR signaling pathway in regulating muscle function, and finally aimed to investigate the interaction between chronic (24 hr) electrical stimulation and leucine supplementation in regulating muscle force. We hypothesized that the addition of leucine would augment muscle size and force production in an mTOR dependent manner and that leucine and electrical stimulation would act together to enhance maximal force in engineered skeletal muscle.

## 2 METHODOLOGY

### 2.1 Cell culture

C2C12 myoblasts were purchased from ECACC, and cultured in growth medium (GM) which consisted of high glucose DMEM (Sigma–Aldrich, Dorset, UK), 20% FBS (FBS Good: PAN Biotech, Aidenbach, Germany) and 1% Penicillin‐Streptomycin solution (GIBCO/Fisher Scientific, Leicestershire, UK). GM was replenished every other day until cells were approximately 80% confluent, at which point they were trypsinized and counted using the trypan blue exclusion method prior to plating on fibrin hydrogels. All experiments were conducted using cells which had undergone fewer than 10 passages.

### 2.2 Tissue engineered skeletal muscle constructs

Fibrin hydrogels were fabricated as previously described (Martin et al., 2015). In brief, two 6 mm silk sutures were pinned into Sylgard coated 35‐mm plates (VWR, Leicestershire, UK) 12 mm apart using 0.15 mm minutien pins (Entomoravia, Slavkov u Brna, Czech Republic). Plates were sterilized by ultraviolet light and washing with 70% ethanol and left to dry for approximately 3 hr. Each plate then received 500 μl of GM containing 10U/ml thrombin (Sigma–Aldrich) and 80 mg/ml aprotinin (Sigma–Aldrich) which was spread evenly over the surface of the plate ensuring the sutures were fully covered. 200 μl of 20 mg/ml stock fibrinogen (Sigma–Aldrich) solution was then added to the plate, and was agitated gently to ensure even distribution and then left to incubate for 10 min at room temperature before being transferred to the incubator (37°C) for 1 hr. Following polymerization of the hydrogels, 1 × 10^5^ C2C12 myoblasts were seeded on to the surface of the gel in 2 ml of GM which contained 0.25 mg/ml 6‐aminocaproic acid (Sigma–Aldrich) to help prevent degradation of the fibrin gel. GM was replenished daily for 3 days, at which point the cells were confluent and the media was switched to differentiation media (DM) consisting of high glucose DMEM containing 2% Horse serum (Sigma–Aldrich), 1% Penicillin‐streptomycin and 0.5 mg/ml 6‐aminocaproic acid. Following 48 hr in DM, media was switched to maintenance media (MM) in accordance with previous reports (Khodabukus & Baar, 2009) consisting of high glucose DMEM, 7% FBS, 1% penicillin‐ streptomycin, and 0.5 mg/ml 6‐aminocaproic acid. MM was changed daily for the duration of the experiment (14 days) and was supplemented with 1, 5, or 20 mM of L‐leucine (Sigma–Aldrich) and/or rapamycin (100 nM; Millipore, Hertfordshire, UK) from day 9 onward for functional, morphological, and mRNA analyses. It is of note that DMEM contains ∼800 μM of L‐leucine and therefore the doses shown throughout represent the supplemented and not total leucine concentration. For acute mTORC1 signaling analysis, cultures were maintained in MM until day 14, at which point leucine was supplemented as described below.

### 2.3 Immunoblotting

Following 14 days in culture, MM was removed from the engineered muscle, which was washed twice in PBS prior to incubation in Hanks balanced salt solution (HBSS, Sigma–Aldrich) for 60 min. HBSS was then removed before incubation with HBSS (Control) or leucine dissolved in HBSS for 30 min, after which treatment solutions were removed and engineered muscle was blotted dry, frozen in liquid nitrogen and stored at −80°C until further analysis. Samples were subsequently homogenized in 200 μl of RIPA lysis buffer (Fisher Scientific) containing a protease and phosphatase inhibitor cocktail (Fisher Scientific) and rotated for 1 hr at 4°C before being centrifuged at 12,000 × *g* in order to remove insoluble material. The supernatant was transferred to a fresh tube and protein concentrations were determined using the Pierce 660 protein assay (Fisher Scientific). Protein was mixed with 4X laemmli buffer (Sigma–Aldrich) and boiled at 95°C for 5 min. Equal volumes of protein (7.5 μg) were loaded in to precast 4–12% gradient SDS‐ polyacrylamide gels (TruPAGE, Sigma–Aldrich) and separated by electrophoresis at 150V. All samples within a single experiment were loaded on to a single gel and duplicate gels were run in order to detect phosphorylated and total proteins. Proteins were transferred on to nitrocellulose membranes (GE healthcare, Fisher Scientific) at 0.2A for 90 min, and blocked in 5% BSA at 4°C for 90 min. Thereafter, membranes were washed three times in tris‐buffered saline + 0.1% tween (TBST) and incubated in primary antibody overnight at 4°C as follows: phospho‐4EBP‐1 (1:1500), total‐4EBP‐1 (1:2000), phospho‐rpS6 (1:2000), total‐rpS6 (1:2000). All antibodies were purchased from Cell Signaling Technology, Massachusetts. Following three further washes in TBST, membranes were incubated for 1 hr at room temperature in HRP‐conjugated anti‐rabbit IgG secondary antibody (Sigma–Aldrich) diluted 1:1500 in TBST containing 5% skimmed milk powder before detection with chemilluminescence. Imaging and band quantification were conducted on a ChemiDoc imaging system (Bio‐rad, Hertfordshire, UK) using Quantity One image software (Version 4.6.8, Bio–rad). Phosphorylation levels are expressed relative to total protein and α‐tubulin (1:2000, Cell Signaling Technology) abundance, and are presented as a fold change compared to a single control sample in each experiment.

### 2.4 RNA extraction and RT‐qPCR

Following 5 days of incubation with Control, 1 mM, 5 mM, or 20 mM of Leucine, engineered muscle constructs were washed once in PBS, blotted dry, snap frozen in liquid nitrogen and stored at −80°C for further analysis. Engineered muscles were subsequently homogenized in 500 μl of TRI Reagent (Sigma–Aldrich) and RNA was isolated according to the manufacturer's instructions, and re‐suspended in 50 μl of RNA storage solution (Fisher Scientific). RNA concentration and quality was assessed by UV spectroscopy at optical densities of 260 and 280 nm using a Nanodrop 2000 spectrophotometer (Thermo Fisher, Leicestershire, UK).

RT‐qPCR reactions were conducted in triplicate in 384 well plates and consisted of 20 ng of RNA diluted in 5 μl of nuclease free water, 0.1 μl of both forward and reverse primers at a final concentration of 2 μM (see Tables 1 and S1 for primer sequences), 0.1 μl of Quantifast reverse transcriptase kit (Qiagen, West Sussex, UK) and 4.7 μl of Sybr Green mix (Qiagen). One‐step RT‐qPCR was performed on a Viia7™ thermal cycler (Applied Biosystems/Thermo Fisher), which was programed to perform the following: 10 min at 50°C (reverse transcription), 5 min at 95°C (“Hot Start” Taq polymerase), followed by 40 cycles of 95°C for 10 s and 60°C for 30 s. Fluorescence was detected at the end of each cycle and data were analyzed using the 2^(−ΔΔC^_T_^)^ method (Livak & Schmittgen, 2001) using POLR2B as a reference gene and a single control construct from each experiment as a calibrator.

**Table 1:** Primer sequences used to investigate proteolytic mRNA expression in the present study

mRNA of interest	Primer sequence 5′‐3′	Reference number	Product length
*Trim63*	F: CCAAGGAGAATAGCCACCAG	NM_001039048.2	84
	R: CGCTCTTCTTCTCGTCCAG		
*Fbxo32*	F: CTGAAAGTTCTTGAAGACCAG	NM_026346.3	79
	R: GTGTGCATAAGGATGTGTAG		
*Map1lc3a*	F: AGTTGGTCAAGATCATCCG	NM_025735.3	130
	R: TCATCCTTCTCCTGTTCATAG		
*Gabarap*	F: AATCCGAAAGAAATACCCAG	NM_019749.4	175
	R: GAAAAACAAGGCATCTTCAG		
*Polr2b*	F: GGTCAGAAGGGAACTTGTGGTAT	NM_153798.2	197
	R: GCATCATTAAATGGAGTAGCGTC		

*Trim63*, Muscle Ring Finger‐1; *Fbxo32*, Muscle Atrophy F‐box; *Gabarap*, Gamma‐aminobutyric acid receptor‐associated protein; *Map1lc3a*, Microtubule‐associated protein 1A/1B‐light chain 3. Polr2b, RNA polymerase II polypeptide B.

### 2.5 Immunostaining

Following 5 days of treatment, each engineered muscle was washed with PBS and fixed using ice cold methanol; acetone solution. Subsequently, engineered muscle constructs were cut away from the sutures and placed on poly‐lysine coated microscope slides (VWR, Leicestershire, UK) and ringed with PAP pen (DAKO, Cambridgeshire, UK). Constructs were blocked with 1× Tris buffered saline (TBS; 0.5 M) containing 5% goat serum (Sigma–Aldrich) and 0.2% Triton‐x‐100 (Fisher Scientific) for 90 min. Following three washes with TBS, constructs were incubated overnight in a humidified staining chamber with rabbit polyclonal anti‐desmin primary antibody (Abcam, Cambridgeshire, UK) diluted 1:200 in TBS. After overnight incubation, constructs were washed three times in TBS and incubated for 3 hr with goat anti‐rabbit TRITC secondary antibody (Abcam) diluted 1:200 in TBS, and DAPI (Sigma–Aldrich) in order to visualize nuclei. Following three further washes in distilled water, constructs were mounted on glass coverslips using a drop of Fluoromount™ (Sigma–Aldrich) mounting medium. Engineered muscle constructs were imaged using a Zeiss LSM‐710 confocal microscope (Zeiss, Cambridgeshire, UK) and were analyzed using Image J software (NIH).

### 2.6 Acute functional testing

Functional testing of engineered muscle was conducted as previously described (Martin et al., 2015). Briefly, at the end of the culture period (14 days total/5 days of treatment) engineered muscle constructs were washed twice in PBS and one anchor from the fibrin construct to be analyzed was removed from the sylgard and attached to a model 403A force transducer (Aurora Scientific, Dublin, Ireland) using canning wax, and a micro‐manipulator was used in order to precisely control the position of the engineered muscle. Krebs Ringer Hepes (KRH; 10 mM HEPES, 138 mM NaCl, 4.7 mM KCl, 1.25 mM CaCl_2,_ 1.25 mM MgSO, 5 mM Glucose, 0.05% Bovine Serum Albumin in dH_2_O) buffer solution was added to the dish containing the construct and two stainless steel electrodes were placed in position either side of the construct and submerged in the KRH buffer prior to functional testing. Impulses were generated using LABview software (National Instruments, Berkshire, UK) connected to a custom built amplifier and maximal tetanic contractions were elicited by stimulating at 100 Hz at 3.5 V/mm. Data were acquired using a Powerlab 4/25T unit with associated software (AD instruments, Oxfordshire, UK) with a sampling rate of 1 KHz.

### 2.7 Electrical stimulation

After 13 days in culture, MM was removed from engineered muscle constructs and constructs were transferred to a custom built 3‐D printed plate and 4 ml of respective MM were replenished. A modified 6‐well plate lid with pairs of stainless steel electrodes 1 cm apart was then placed on to the constructs, with care taken to ensure that each set of electrodes were positioned either side of the muscle constructs in order to deliver electric field stimulation. Electrical stimulation was delivered to the constructs using LABview software (National Instruments) connected to a custom built amplifier and in turn attached to the stainless steel electrodes. Based on a previously published protocol (Khodabukus & Baar, 2012) with some slight modifications, stimulation consisted of 5 bipolar 1 ms pulses delivered at 1 V/mm and 10 Hz with 3.5 s rest periods. After 24 hr, stimulation was terminated and constructs were tested as described above in section 2.6.

### 2.8 Statistical analysis

All data are presented as mean ± SEM. Normality of distribution and homogeneity of variance in all data sets were determined using a Shapiro–Wilk test and Levene's tests, respectively. Data were subsequently analyzed using either One–Way ANOVA with Tukey HSD post–hoc tests or Kruskall Wallis tests where data were not normally distributed. All analysis was conducted using SPSS version 22.

## 3 RESULTS

### 3.1 Leucine induces dose‐dependent phosphorylation of downstream mTORC1 targets, but does not affect proteolytic mRNA expression in engineered skeletal muscle

**Figure 1:**
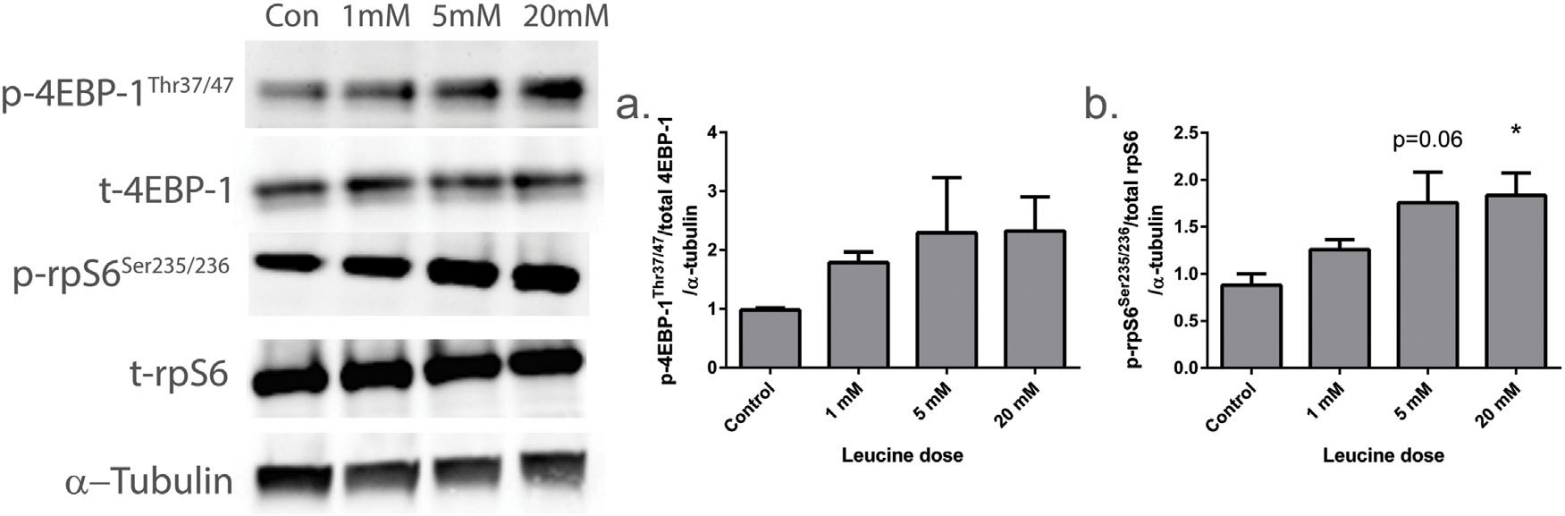
Induction of mTORC1 signaling following incubation of tissue engineered skeletal muscle with increasing doses of leucine. (a) 4EBP‐1^Thr37/47^ phosphorylation is increased as the leucine concentration in elevated, although this effect did not reach statistical significance. (b) rpS6^Ser235/236^ phosphorylation increased in a dose dependent manner and was only significantly elevated above control with the addition of 20 mM of leucine. Data are mean ± SEM for a minimum of *n* = 4 engineered muscles. * indicates statistically greater than control (*p* < 0.05)

Since, acute supplementation with leucine has been shown to activate mTORC1, initial experiments aimed to see if this effect was also apparent in engineered muscle, and if it was dose dependent. Phosphorylation of 4EBP‐1^Thr37/46^, a regulator of cap‐dependent translation appeared to increase in response to leucine, although this effect did not reach statistical significance (*p* = 0.07, Figure 1a). Ribosomal protein S6 (rpS6^Ser235/236^) phosphorylation, was also elevated in response to leucine in a dose dependent manner (0.88 ± 0.12, 1.25 ± 0.11, 1.76 ± 0.33, 1.84 ± 0.24 in control, 1 mM, 5 mM, and 20 mM groups, respectively; *p *< 0.05), whereby only supplementation with 20 mM of leucine was sufficient to induce a statistically significant increase in phosphorylation above control (Figure 1b).

To determine if leucine had any impact on proteolytic pathways, mRNA expression of markers of the autophagy‐lysosome (*Map1lc3a* and *Gabarap*) and ubiquitin‐proteasome (*Trim63* and *Fbxo32*) systems were measured (Table 2). Five days of leucine supplementation at increasing doses had no impact on *Trim63* (*p* = 0.88) or *Fbxo32* (*p* = 0.47) mRNA expression, or the levels of *Gabarap* (*p* = 0.88) or *Map1lc3a* (*p* = 0.07), although, the latter approached significance through the observed increase in expression seen with 20 mM leucine supplementation.

**Table 2:** Proteolytic mRNA expression following 5 days of incubation of tissue engineered skeletal muscle with increasing doses of leucine

	Control	1 mM Leu	5 mM Leu	20 mM Leu	*p*‐value
*Trim63*	0.94 ± 0.11	0.87 ± 0.13	0.90 ± 0.12	0.93 ± 0.14	0.81
*Fbxo32*	1.10 ± 0.07	1.19 ± 0.06	1.07 ± 0.03	1.10 ± 0.05	0.47
*Map1lc3a*	1.22 ± 0.18	1.11 ± 0.02	1.09 ± 0.07	1.60 ± 0.28	0.07
*Gabarap*	1.07 ± 0.06	1.08 ± 0.03	1.05 ± 0.01	1.09 ± 0.04	0.88

Data are expressed as mean ± SEM for *n* = 4 engineered muscles.

### 3.2 Leucine supplementation augments myotube size and contractile force in tissue engineered skeletal muscle

**Figure 2:**
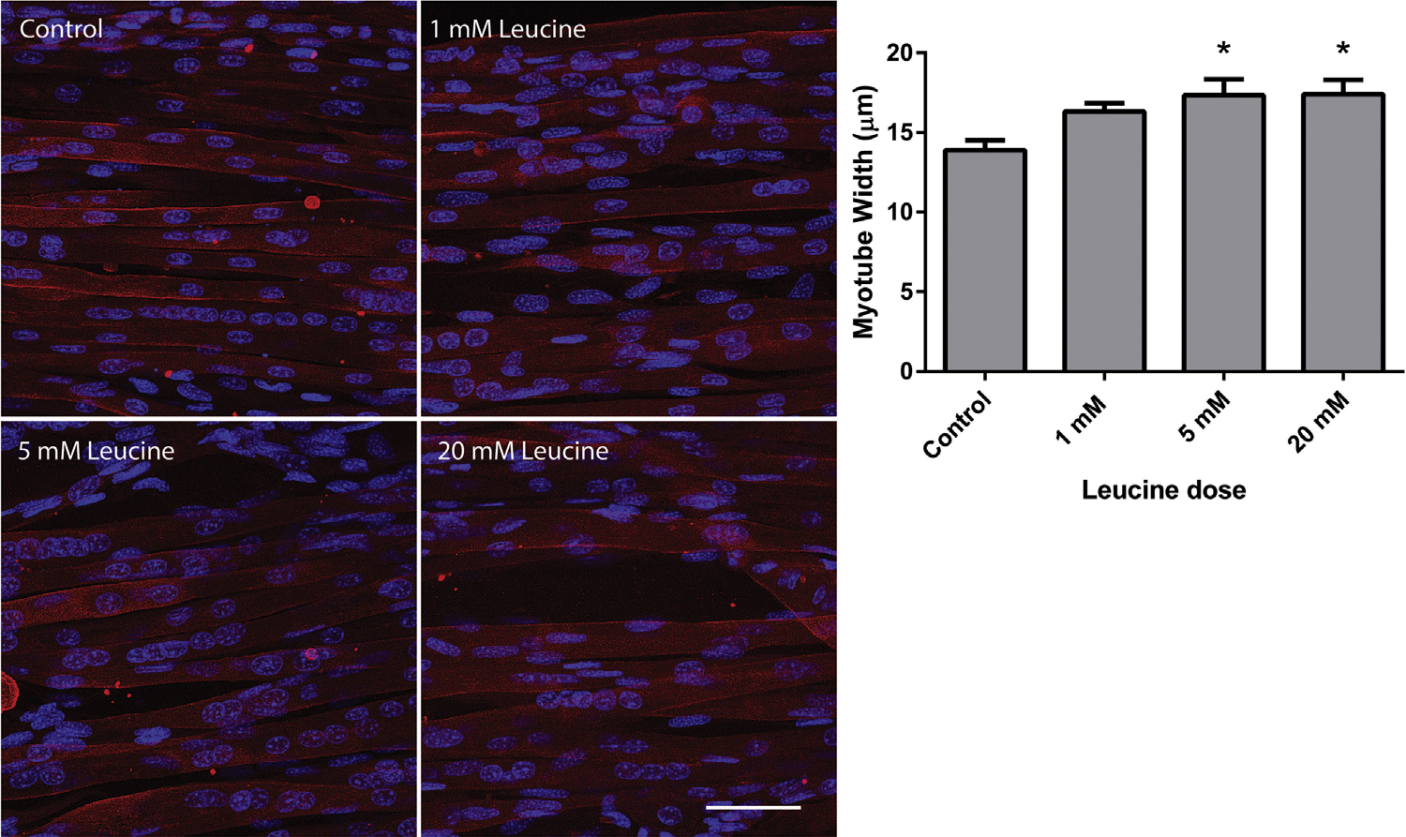
Myotube hypertrophy following 5 days of supplementation of engineered skeletal muscle with increasing doses of leucine. Desmin staining (red) of myotubes was significantly (*p* < 0.05) increased with 5 and 20 mM of leucine supplementation. Myotubes are counterstained with DAPI (blue) and scale bar indicates 50 µm. Data are expressed as mean ± SEM for *n* = 5 engineered muscles. * indicates statistically greater than control (*p* < 0.05)

Leucine had a hypertrophic effect on engineered muscle, as evidenced by the increase in myotube width in supplemented constructs compared with controls. All doses of leucine appeared to result in myotube growth (Figure 2), with myotube width in constructs supplemented for 5 days with 1 mM leucine measured at 16.3 ± 1.2 μm, 5 mM at 17.3 ± 2.3 μm, and 20 mM at 17.4 ± 1.8 μm, compared to control constructs where myotube width was measured at 13.9 ± 1.4 μm after 14 days in culture. In this instance, while the mean increase in myotube width was apparent compared to controls even with 1 mM leucine supplementation, this was not significant (*p* = 0.15), and therefore more than 1 mM leucine was required to induced significant hypertrophy in engineered skeletal muscle.

**Figure 3:**
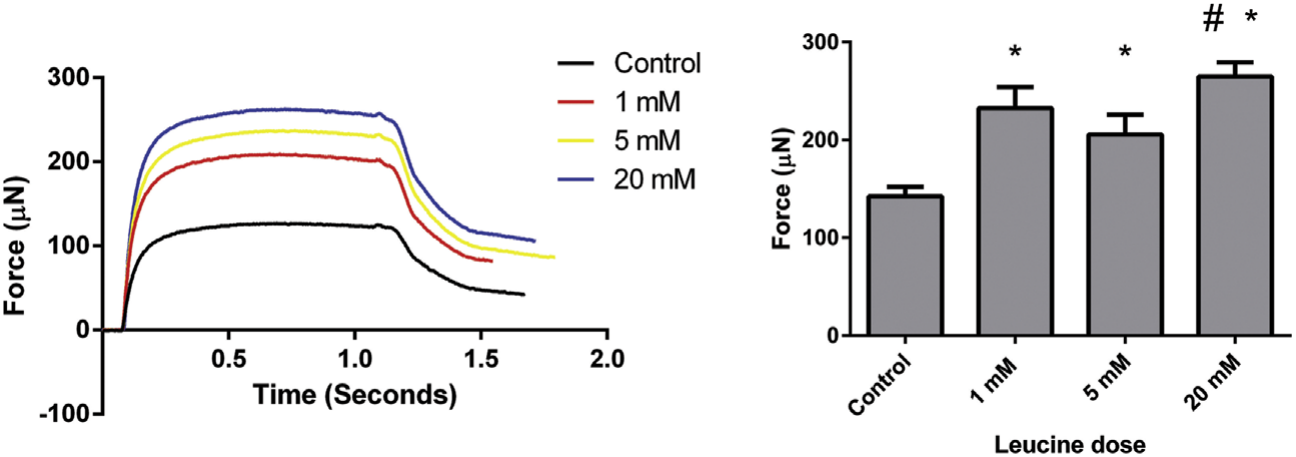
Leucine supplementation increases contractile force in engineered skeletal muscle independent of dose. Maximal contractile force was enhanced in engineered constructs supplemented with leucine for 5 days at the end of the culture period. Data are expressed as mean ± SEM for *n* = 5 engineered muscles. * indicates statistically greater than control (*p* < 0.05), ^#^ indicates statistically greater than 5 mM (*p* < 0.05)

C2C12 engineered muscles predominantly expressed type I myosin heavy chain isoforms and exhibited positive force frequency when stimulated (Figure S1), thus confirming engineered muscle as a suitable model of adult skeletal muscle; and thus the effects of leucine on in vitro muscle function were determined. The addition of leucine to the cell culture media significantly enhanced relative force production (*p* < 0.05), with all three concentrations associated with elevated tetanic force production compared to the control constructs (Figure 3). Interestingly, although the addition of leucine augmented force production, this did not appear to be dose‐dependent, with 1, 5, and 20 mM leucine concentrations augmenting mean maximal force by 63.5%, 44.5%, and 86.3%, respectively in comparison to control constructs, with the difference between 5 mM and 20 mM reaching statistical significance (*p *< 0.05).

### 3.3 Enhanced contractile force with leucine supplementation is mTOR dependent

**Figure 4:**
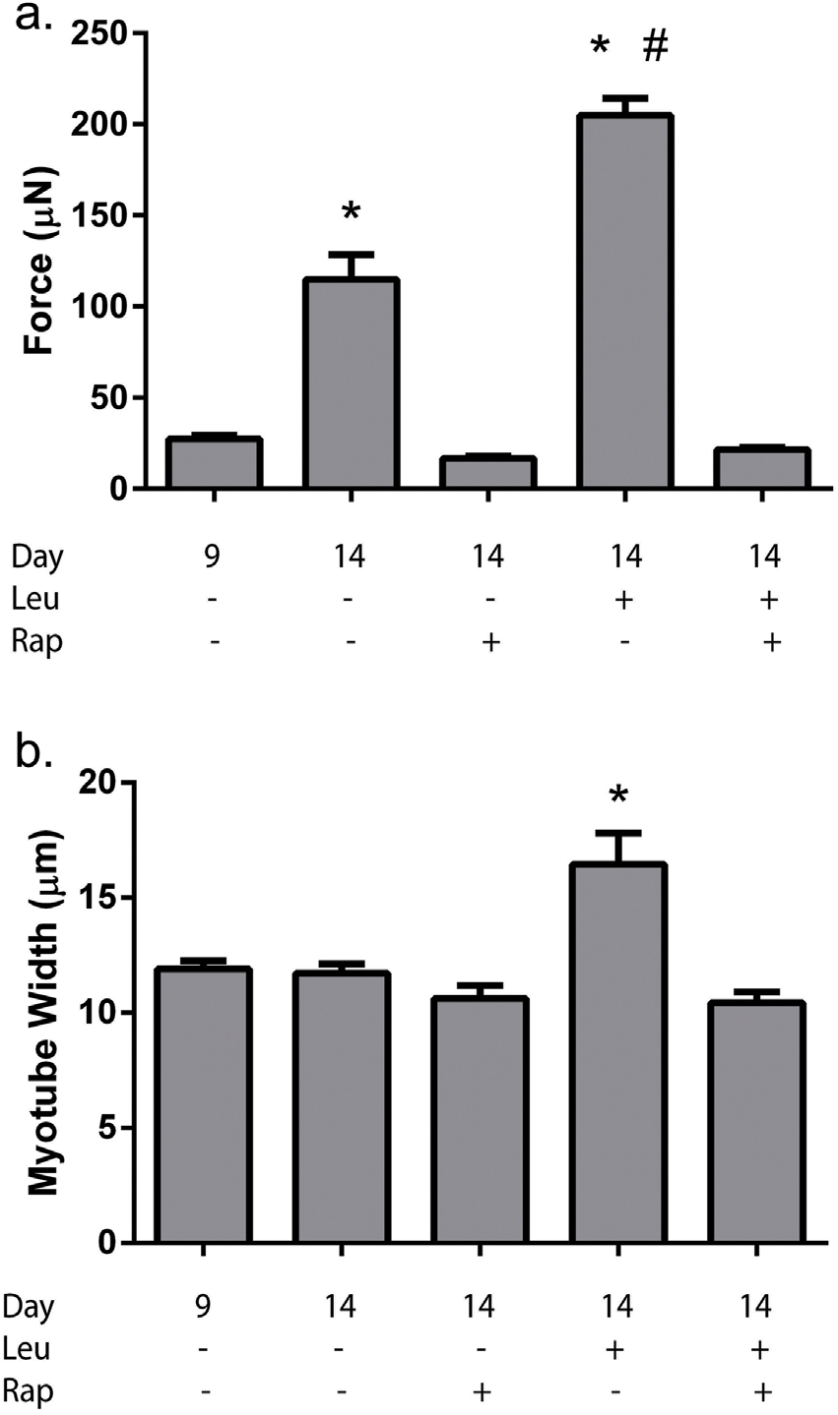
Leucine associated increases in contractile force and myotube size are mTOR dependent. (a) Addition of the mTOR inhibitor rapamycin (100 nM) for the final 5 days of culture either alone or in combination with leucine (20 mM) resulted in blunted maximal force production which was similar to that seen at day 9 of culture and lower than that observed after 14 days of culture. (b) Rapamycin prevented the leucine induced myotube hypertrophy, but did not induce significant atrophy of the myotubes. Data are mean ± SEM for a minimum of *n* = 4 engineered muscles. * indicates statistically greater than 9 day Control (*p* < 0.05), ^#^ indicates significantly greater than 14 day control (*p* < 0.05)

To test whether the increase in force associated with leucine supplementation was mTOR dependent, leucine was next co‐incubated with the mTOR inhibitor rapamycin (see supplementary Figure S2). We tested engineered skeletal muscle under five conditions, namely; day 9 control (the time at which leucine was added), day 14 control, rapamycin alone, leucine alone, and leucine + rapamycin. Engineered muscle supplemented with leucine again produced greater contractile force relative to 14 day old control constructs (204.8 ± 9.4 μN vs. 114.8 ± 13.5 μN, *p* < 0.05). When leucine was supplemented in combination with 100 nM rapamycin however, the increase in force was completely blunted. Indeed, the addition of rapamycin either alone (16.7 ± 1.4 μN) or in combination with leucine (21.6 ± 1.1 μN) resulted in maximal contractile force lower than that of 14 day old controls but similar to that of day 9 controls (27.5 ± 1.9 μN, Figure 4a).

To determine if the blunting of contractile force was driven by attenuated myotube hypertrophy, engineered muscles were stained for desmin and myotube widths determined. As expected, co‐incubation of leucine with rapamycin completely blocked hypertrophy, with average myotube widths in leucine supplemented constructs measuring 16.5 ± 3.0 μm compared to 10.5 ± 1.0 μm in the leucine + rapamycin engineered muscles (*p* < 0.05), while no difference existed in myotube width between either day 9 and 14 controls or rapamycin alone or in combination with leucine (Figure 4b). In addition, there was no difference in the number of myotubes between conditions (supplementary Figure S2). This suggests that rapamycin completely attenuates leucine associated myotube hypertrophy, and furthermore shows that the increase in contractile force from day 9 to 14 in control constructs is not related to increased myotube width.

### 3.4 Leucine in combination with contractile activity cumulatively improve muscle function

**Figure 5:**
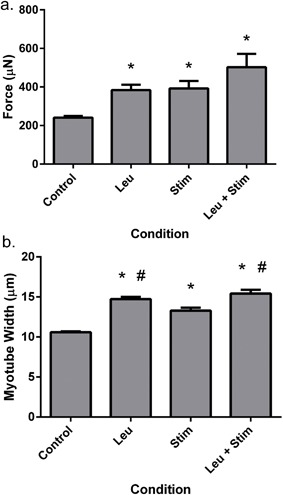
Combination effects of leucine and electrical stimulation on engineered skeletal muscle function and myotube size. (a) Leucine and electrical stimulation augment maximal force production independently, and in combination further increase force production above control. (b) myotube growth is enhanced by both electrical stimulation and leucine above control, while leucine appears to have the greater effect on myotube size overall. Data are mean ± SEM from a minimum of *n* = 4 engineered muscles. * indicates statistically greater than control (*p* < 0.05), ^#^ indicates statistically greater than stimulation alone (*p* < 0.05)

Since, both muscle loading and amino acids have the capability to enhance muscle size in vivo, it was next asked whether a combination of leucine supplementation and muscle contraction can additively increase muscle function in vitro. Maximal contractile force was significantly enhanced by leucine supplementation (383.8 ± 27.6 μN), electrical stimulation (392.6 ± 38.5 μN) and a combination of leucine and electrical stimulation (502.4 ± 69.3 μN) compared to control engineered skeletal muscle (240.8 ± 8.7 μN, *p* < 0.05). Moreover, while the effects of leucine (59.4% increase) and electrical stimulation (63.0% increase) augmented force to a similar extent), the effects of the two stimuli in combination resulted in maximal contractile force higher than either stimulus in isolation (108.6% increase, Figure 5a), although this did not reach statistical significance (leucine vs. leucine + Stimulation, *p* = 0.06).

Interestingly, subsequent immunocytochemical analysis revealed that myotube width was increased in response to electrical stimulation (13.3 ± 0.7 μm), leucine (14.7 ± 0.6 μm) and stimulation plus leucine in combination (15.4 ± 0.9 μm) compared to control (10.6 ± 0.2 μm, *p* < 0.05). Myotube width was significantly greater in leucine supplemented engineered constructs and leucine plus electrical stimulation constructs compared to electrical stimulation alone (*p *< 0.05), however no difference was apparent between leucine alone and in combination with electrical stimulation (*p* = 0.484, Figure 5b).

## 4 DISCUSSION

Ingestion of amino acids and particularly the branched chain amino acid leucine has been shown to be capable of activating acute anabolic intracellular signaling and MPS (Apro et al., 2015; Churchward‐Venne et al., 2012; Moberg et al., 2014), however there are little data which have examined the more chronic effects of leucine on skeletal muscle mass and function, largely due the lack of appropriately controlled experimental model. In the present study, we used tissue engineered skeletal muscle to determine if leucine could enhance contractile function and drive muscle hypertrophy in vitro. Our data suggest that leucine activates mTORC1 signaling, and augments muscle size and function in engineered muscle and that this improvement in maximal force production is mTOR sensitive. We also found that both leucine and chronic muscle contraction are capable of increasing muscle force, and together result in greater functional enhancement than either stimulus in isolation.

It is well recognized that mTORC1 activation leads to elevations in translation initiation and ribosome biogenesis, in turn enhancing MPS and capacity for cellular growth. We show here in engineered skeletal muscle, that leucine activates the downstream effectors of mTORC1, namely 4EBP‐1 and rpS6, confirming previous studies conducted both in conventional in vitro culture and in vivo (Anthony et al., 2000; Areta et al., 2014; Atherton, Smith et al., 2010; Churchward‐Venne et al., 2012), and thus providing strong validation for the use of engineered muscle for investigations in this area. Interestingly, we also found that this effect on mTORC1 signaling appears to be dose dependent, with higher doses (5–20 mM) required in order to maximize the response. This is somewhat in agreement with Areta et al. (2014) who found a dose dependent increase in p70S6 kinase phosphorylation with leucine supplementation, although, this was not mirrored in other mTORC1 related protein kinases tested. Furthermore, in the present study we found that 5 days of leucine supplementation had no effect on the expression of markers of the ubiquitin‐proteasome or autophagy‐lysosome system. This was slightly surprising since mTORC1 activation is associated with inhibition of autophagy (Sandri, 2013), and there is evidence that amino acid ingestion can also prevent elevations in MuRF‐1 (*Trim63*) and MAFbx (*Fbxo32*) expression at rest and following exercise (Borgenvik, Apro, & Blomstrand, 2012; Herningtyas et al., 2008). Our in vitro data support in vivo human data reporting no alterations in proteolytic gene expression following ingestion of leucine or its metabolite β‐Hydroxy‐β‐methylbutyrate in healthy young men (Wilkinson et al., 2013), although, since we measured changes in mRNA expression, we cannot discount the possibility that differences may have been observed at an earlier time point.

Myotube growth following chronic leucine supplementation followed a similar dose‐dependent trend to that of the mTORC1 signaling induction. Indeed, although myotube hypertrophy was observed with 1 mM leucine supplementation, this effect was not statistically significant until higher doses were supplemented. Furthermore, this concordance between the anabolic signaling response and the alteration in myotube hypertrophy supports the work of (Mitchell et al., 2014), which showed a positive relationship between 4EBP‐1 phosphorylation and changes in muscle volume with resistance training in men, suggesting that the responses observed in vivo are closely mirrored in engineered skeletal muscle in vitro.

A key finding of our study was that supplementing the culture media of engineered skeletal muscle constructs with leucine for the final 5 days of experimentation augmented maximal contractile force by up to ∼80%. Indeed, while other investigations have observed an augmented functionality of engineered muscles following chronic treatment with pharmacological agents (Madden, Juhas, Kraus, Truskey, & Bursac, 2015; Syverud, VanDusen, & Larkin, 2016; Weist et al., 2013), this represents the first report of how amino acids can enhance skeletal muscle contractile function in vitro, thus providing novel data regarding leucine's anabolic properties. Furthermore, the present data show that the enhancement in maximal force production in the presence of leucine was not dose‐dependent, which is in contrast to our findings for signaling through mTORC1 and myotube size, and perhaps suggests that the additional muscle growth at higher leucine doses is partially a result of accretion of non‐contractile proteins.

Rapamycin completely blunted the increase in force which was found to occur over the final 5 days of culture (day 9–14) in the absence of leucine, suggesting that this adaptation is mTOR dependent. Moreover, since neither myotube size or total number were reduced with rapamycin treatment, and were not different over the final 5 days of culture, the increase in force is likely due to other mTOR dependent processes leading to maturation of the myotubes. Indeed, in mice where mTOR is specifically knocked out in skeletal muscle maximal force production was reduced even when accounting for the loss of muscle size, and this force decrement was associated with reduced expression of components of the dystrophin‐dystroglycan complex (Risson et al., 2009). While this was not a primary outcome of the present study, it would be interesting in the future to determine the reasons for blunted force production in engineered skeletal muscle treated with rapamycin.

When rapamycin was incubated alongside leucine the measured contractile force was equivalent to that measured at 9 days of culture (i.e., less than that observed at day 14 in the absence of leucine), clearly showing that leucine augments contractile force in an mTOR sensitive manner. Rapamycin also completely blunted leucine associated myotube growth, similar to previous findings in rodents following compensatory hypertrophy (Bodine et al., 2001), suggesting that the impact on muscle force was at least partially due to lack of muscle growth.

Since, muscle loading (e.g., resistance exercise) and amino acids can stimulate mTORC1 and protein synthesis through diverse mechanisms (Marcotte et al., 2015), and as such implement an additive effect on muscle growth, we conducted a final set of experiments to determine if this effect could be modelled in vitro, and whether the two stimuli would act together to increase maximal muscle force production. It was found that both leucine and electrical stimulation alone resulted in an approximately 60% increase in maximal force production, while combining the two stimuli resulted in an approximately 110% increase in contractile force compared to control muscles. The augmentation of maximal contractile force of engineered skeletal muscle described here is pertinent for the tissue engineering community, since a considerable limitation in the use of engineered muscle constructs for regenerative medicine lies in the fact that force production is far less than that of native muscle (Bian & Bursac, 2008). As such, the present data reveals that the use of electrical stimulation in combination with leucine may help to overcome this limitation, and when used in combination with other factors such as TGF‐β or agrin, which have previously been shown to enhance contractile force (Bian & Bursac, 2012; Weist et al., 2013) may allow the use of engineered muscle for regenerative medicine to become a reality.

While electrical stimulation alone also resulted in myotube hypertrophy, in our system, and with the variables tested, leucine appears to be a greater driver of muscle growth since leucine alone resulted in greater hypertrophy than stimulation alone and was not different to leucine and stimulation in combination. This may be a result of the discrepancy between respective intervention times (i.e., 24 hr stimulation vs 5 days leucine), and it is of note that the stimulation regime was chosen based on a protocol known to enhance contractile force. Khodabukus & Baar (2012) have previously established that the augmentation in force as a consequence of the same 24 hr electrical stimulation as used in the present study can only be ablated by approximately 40% in the presence of rapamycin, suggesting that the additional increase in force is driven through alternative mechanisms and not myotube hypertrophy. In the future it would be interesting to see if longer periods of contractile activity result in increased myotube growth, and whether the effect of leucine remains additive.

In conclusion, the present work shows that the amino acid leucine can activate mTORC1 signaling and muscle growth in tissue engineered skeletal muscle and that this response appears to be somewhat dose dependent. Importantly, we show for the first time that leucine can enhance maximal contractile force in an mTOR‐sensitive manner and that leucine and electrical stimulation can be used together to augment this response. These data provide strong validation for the use of engineered skeletal muscle as a tool in biomedical research concerned with nutrition and skeletal muscle physiology and function, and highlights the potential for leucine supplementation as a clinical therapy in conditions associated with impaired muscle strength and reduced size.

## Supporting information

Additional Supporting Information may be found online in the supporting information tab for this article.

**Figure S1**. Indicators of development and maturation in engineered skeletal muscle. (a) Myosin Heavy Chain mRNA expression in engineered muscle at 14 days of culture shows an abundance of MYH1 transcription, suggesting the development of an adult phenotype. Corresponding protein is shown in brackets. (b) Engineered skeletal muscle is capable of producing contractile force and shows positive force‐frequency after 14 days in culture. Data are expressed as mean ± SEM.

**Figure S2**. Activation of mTORC1 signalling with leucine is inhibited by co‐incubation with 100nM rapamycin. Myotubes were starved for 60 minutes, and rapamycin was added for the final 30 min before being incubated for 30 min as indicated.

**Figure S3**. Myotube density per microscope field is not affected by the addition of leucine or rapamycin to the culture media for the final 5 days of experimentation. Data are mean ± SEM.

**Table S1**. Primer sequences used to investigate Myosin Heavy Chain mRNA expression in the present study.
