# Supplementary figures and images for: Leucine elicits myotube hypertrophy and enhances maximal contractile force in tissue engineered skeletal muscle in vitro
Source: J Cell Physiol. 2017 May 8;232(10):2788–97. doi: 10.1002/jcp.25960 (PMC5518187; doi:10.1002/jcp.25960)

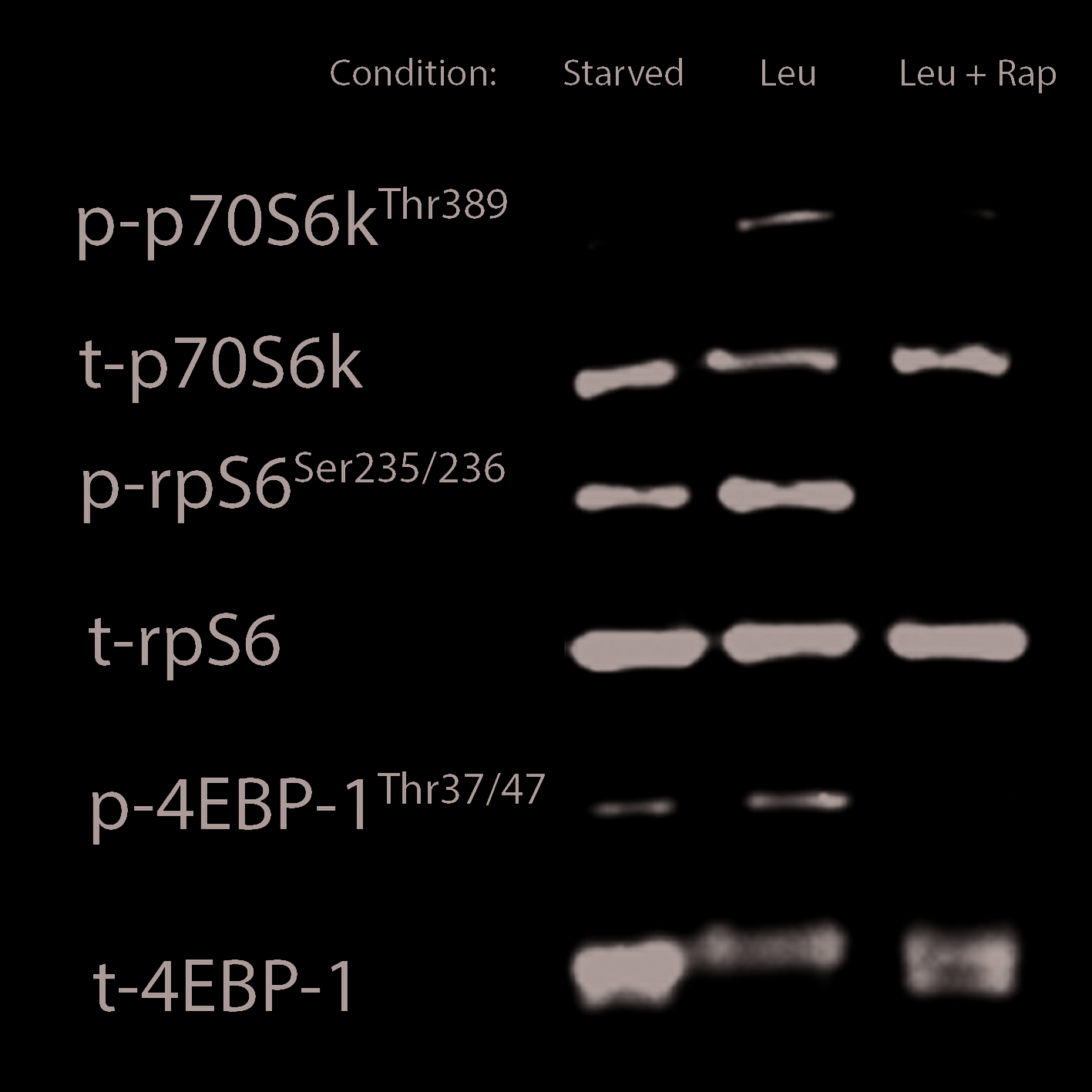

Supplement: Supplementary file 2 — Figure S2. Activation of mTORC1 signalling with leucine is inhibited by co‐incubation with 100nM rapamycin. Myotubes were starved for 60 minutes, and rapamycin was added for the final 30 min before being incubated for 30 min as indicated. [file JCP-232-2788-s002.tif]

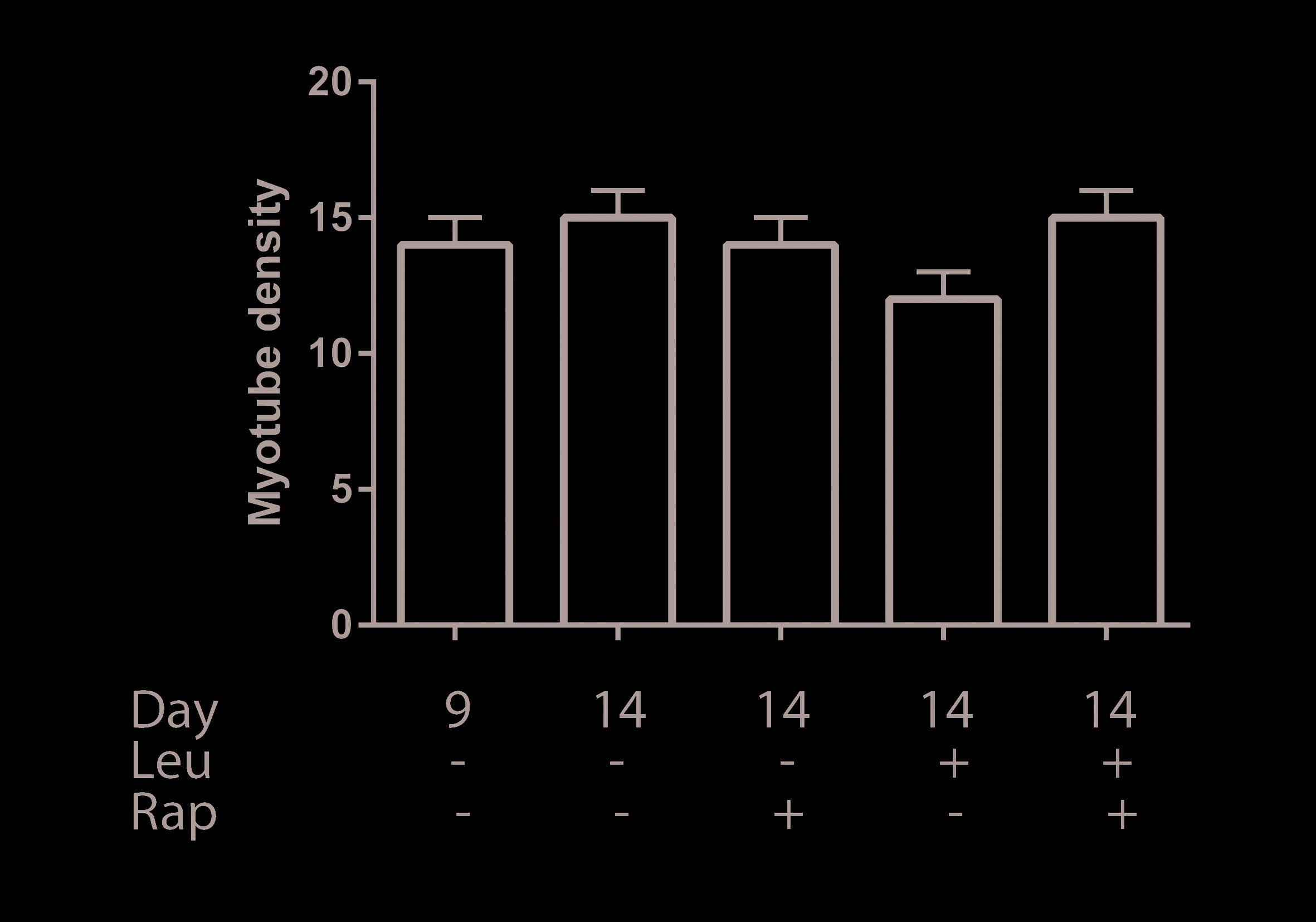

Supplement: Supplementary file 3 — Figure S3. Myotube density per microscope field is not affected by the addition of leucine or rapamycin to the culture media for the final 5 days of experimentation. Data are mean ± SEM. [file JCP-232-2788-s003.tif]
