# Supplementary material for: Leucine elicits myotube hypertrophy and enhances maximal contractile force in tissue engineered skeletal muscle in vitro
Source: J Cell Physiol. 2017 May 8;232(10):2788–97. doi: 10.1002/jcp.25960 (PMC5518187; doi:10.1002/jcp.25960)
Supplement: Supplementary file 4 — Table S1. Primer sequences used to investigate Myosin Heavy Chain mRNA expression in the present study. [file JCP-232-2788-s004.docx]

Supplementary Table S1. Primer sequences used to investigate Myosin Heavy Chain mRNA expression in the present study.

| mRNA of interest | Primer sequence 5’-3’ | Reference number | Product length |
| --- | --- | --- | --- |
| *Myh1* | F: CGCTTTGGTAAGTTCATCAG  R: TAGATCCGGCTTCTTGTTAG | NM_030679.1 | 165 |
| *Myh2* | F: CAGTTCATGCTAACAGACAG  R: TTGTTGCAAAGTACTGGATG | NM_001039545.2 | 106 |
| *Myh3* | F: CATATCAGAGTGAGGAGGAC  R: CTTGTAGGACTTGACTTTCAC | NM_001099635.1 | 86 |
| *Myh4* | F: TCAAATTATCAGTGCCAACC  R: ACTTCTCTAGCAGATAGGTTTC | NM_010855.3 | 158 |
| *Myh7* | F: GATGATCTATACCTACTCGGG  R: TGATGAGGATGGACTGATTC | NM_080728.2 | 194 |
| *Myh8* | F: TGGATGATCTATACCTACTCAG  R: TTGTCAGAGATGGAGAAGATG | NM_177369.3 | 146 |

*Myh1* = MyHC IIx; *Myh2* = MyHC IIa; *Myh3* = MyHC Embryonic; *Myh4* = MyHC IIb; *Myh7* = MyHC I; *Myh8* = MyHC Neonatal.
